# SGLT-2 Inhibitors—a Culprit of Diabetic Ketoacidosis Postbariatric Surgery

**DOI:** 10.1155/2020/8817829

**Published:** 2020-11-05

**Authors:** Qasim Zafar Iqbal, Danil Mishiyev, Muhammad Raphay Niazi, Zeeshan Zia, Saud Bin Abdul Sattar, Abdullah Jahanghir, Shahed Quyyumi

**Affiliations:** Staten Island University Hospital, Northwell, USA

## Abstract

Sodium-glucose cotransporter-2 SGLT2 inhibitors are antihyperglycemic drugs that are currently being recommended as second-line therapy for patients with diabetes mellitus. They have grown increasingly popular over recent years, as they have been shown to have some protective effects on the heart and kidneys, both organ systems that diabetes mellitus has shown to have deleterious effect on over time. Despite their growing popularity, they have been found to increase the risk of euglycemic diabetic ketoacidosis (DKA). There is an increasing body of literature detailing cases of euglycemic DKA after bariatric surgery. We present a case series of three cases of euglycemic DKA postbariatric surgery in patients with an underlying history of type 2 diabetes mellitus, who were being treated with SGLT2 inhibitors prior to the surgery. All three patients reported to the emergency room with signs, symptoms, and clinical findings of euglycemic DKA. The AACE recommends SGLT2 inhibitors to be discontinued at least 24 hours prior to surgery and resumed when a patient resumes a normal diet. Our patients presented with euglycemic DKA after bariatric surgery, and we recommend more research should be done targeted at the prolonged postoperative course of patients on SGLT-2 inhibitors and into creating specific guidelines for their use after bariatric surgery.

## 1. Introduction

Sodium-glucose cotransporter-2 (SGLT2) inhibitors are antihyperglycemic drugs that are part of a group of drugs that are being recommended as second-line therapy for patients with diabetes mellitus. [1] They have grown increasingly popular over recent years as they have been shown to have some protective effects on the heart [2, 3] and kidneys [4], both organ systems that diabetes mellitus has a deleterious effect on over the course of time. However, despite their growing popularity, they have been found to increase the risk of euglycemic diabetic ketoacidosis (DKA) [5]. Here, we present three cases of euglycemic diabetic ketoacidosis in patients on SGLT-2 inhibitor after they underwent bariatric surgery.

## 2. Case Presentation 1

A 56-year-old male with a pertinent medical history of type 2 diabetes mellitus for the last 20 years, dyslipidemia, GERD, and morbid obesity, four days status post (s/p) reversal of lap band and conversion to Roux-en-Y gastric bypass surgery presented to the emergency department with generalized, constant, deep aching, moderate abdominal pain. It was associated with generalized weakness, malaise, polydipsia, polyuria, and shortness of breath with ambulation. He was started on canagliflozin 300 mg daily a few months prior because his blood sugar was not controlled despite being on metformin 500 mg twice daily.

The patient had Roux-en-Y bypass surgery four day days prior to admission. The surgery was performed without any complications. On discharge, the patient was tolerating a clear liquid diet and was given specific instructions on how to advance it further. The patient was told to resume his home medications and asked to hold his daily dose of basal insulin, with a plan for endocrinology follow-up within a week.

On presentation to the ER, he was tachycardic and tachypneic. The initial laboratory evaluation revealed sodium of 127 mmol/L, potassium of 4 mmol/L, bicarbonate of 4 mmol/L, an elevated creatinine of 1.7 mg/dL, and blood glucose of 208. The anion gap on presentation was 32, and the beta-hydroxybutyrate level came back as greater than 4.5 mmol/L. The initial venous blood gas done showed a pH of 6.91 and a lactate of 2.4 mmol/L. CT scans of the chest and abdomen came back negative for any acute infectious pathology. Initial EKG showed sinus tachycardia and the initial cultures sent but did not show any growth. The patient was admitted to the ICU and started on intravenous insulin infusion, fluids, and dextrose. He was maintained on these intravenous infusions for the next 72 hours till the gap was closed. On closing of the high anion gap, the patient was switched to Insulin Levemir 38 units to be taken at bedtime, and his canagliflozin was discontinued. He was scheduled for an endocrinology follow-up within a week of discharge.

### 2.1. Case Presentation 2

The second case was a 59-year-old female with a pertinent medical history of type 2 diabetes mellitus, hypertension, dyslipidemia, GERD, and morbid obesity s/p laparoscopic sleeve gastrectomy who presented to us with decreased appetite. The surgery was performed 5 weeks prior to her presentation to the emergency room with complaints of inability to tolerate oral intake. She felt an immediate pressure like sensation with any oral intake leading to an aversion to food. It was associated with nausea, flatus, and watery bowel movements. The home medications included dapagliflozin 10 mg daily, which she resumed after the surgery while her home dose of insulin (Lantus 65 units twice daily) was held post-op. The patient also switched to a bariatric pureed diet one week before admission.

In the emergency room, the labs showed a sodium of 136 mmol/L, potassium of 3.6 mmol/L, bicarbonate of 10 mmol/L, a creatinine of 1.0 mg/dL, and blood glucose of 173. The initial calculated anion gap was 32 and the beta-hydroxybutyrate level came back as greater than 9 mmol/L. The venous blood gas showed a pH of 7.28 and a lactate of 0.8. Urinalysis showed that glucose level was more than 1000 mg/dL and a large number of ketones were present in the urine. Initial EKG and the values of the cardiac enzymes were unremarkable. CT scan of the abdomen and pelvis with oral contrast showed no evidence of acute intra-abdominal pathology. A GI series was done considering the patient's symptoms; however, it showed no evidence of leak or obstruction. Similarly, the gastric emptying study was performed to rule out gastroparesis, and it showed normal progressive emptying. The patient was admitted to the ICU and was treated there for 6 days. She was started on intravenous insulin infusion, fluids, and dextrose while in the ICU. The SGLT-2 inhibitor was discontinued, and the patient was ultimately discharged home to be carefully followed up by the endocrinologist.

### 2.2. Case Presentation 3

A 52-year-old female with a pertinent medical history of type 2 diabetes mellitus, hypertension, dyslipidemia, and morbid obesity s/p gastric sleeve bypass surgery performed 2 weeks ago at an outside hospital presented to the emergency room with complaints of feeling tired over the last few days. She also admitted to shortness of breath on exertion, chills, generalized weakness, decreased appetite, and chronic polyuria. She acknowledged in the emergency room that some of these symptoms (decreased appetite, generalized weakness) started when canagliflozin 300 mg daily was initiated 2 years ago. She was also taking metformin 500 mg twice daily for her chronic type 2 diabetes mellitus.

The initial laboratory values showed a sodium of 142 mmol/L, potassium of 3.2 mmol/L, bicarbonate of 8 mmol/L, a creatinine of 1.1 mg/dL, and blood glucose of 196. The calculated anion gap was 35, and her beta-hydroxybutyrate was >9 mmol/L. The initial arterial blood gas showed a pH of 7.20 and a lactate of 1.6. Urinalysis showed that glucose level was more than 1000 mg/dL, and a large number of ketones were present in the urine. CT angiography was done to rule out pulmonary embolism. Initial EKG and the values of the cardiac enzymes were unremarkable. The blood culture done was negative for any urinary tract infection. The patient was admitted to the ICU where she was treated with intravenous fluids, insulin, and dextrose. The patient slowly recovered, and her appetite also improved after which she was able to tolerate oral feeds as well. She was discharged home on subcutaneous insulin. On discharge, the patient was switched to basal-bolus insulin (23 units of Lantus at night and 8 units of lispro with meals). The SGLT-2 inhibitor and metformin were both discontinued, and the patient was requested to follow up with the endocrinologist as an outpatient within a week of discharge.

## 3. Discussion

SGLT-2 inhibitors work by decreasing the reabsorption of glucose in the kidney, hence increasing the glucose excretion and decreasing the glucose levels in the body. They have been found to have benefits in both cardiovascular mortality and kidney protection recently [2–4]. They are currently recommended as a second-line oral agent for type 2 diabetes mellitus (T2DM) and are even seeing a push for becoming the first-line agent in patients with high cardiovascular risk [6, 7]. SGLT-2 inhibitors are known to have several side effects such as an increased number of infections involving the urinary tract, osteoporosis, and euglycemic DKA.

Euglycemic DKA is commonly identified with severe ketoacidosis, bicarbonate less than 10 mEq/L, and blood glucose usually within or around normal limits [5]. The blood glucose in other cases of DKA is usually significantly elevated, and it is this discrepancy that makes euglycemic DKA dangerous because it can easily be missed by clinicians. The precipitating factors for euglycemic DKA are very much similar to those of DKA and would include recent surgery, severe infections, myocardial infarction, stroke, prolonged fasting, vigorous physical activity, and other physical stressors [8]. The specific mechanism by which euglycemic DKA is provoked is still not entirely certain, but there are theories that the glucosuria can cause decreases in insulin release and decrease in renal sodium reabsorption which causes increased ketone reabsorption and increased glucagon release from the pancreas leading to a state of increased ketone production [9].

There is a growing body of literature on patients that were on SGLT-2 inhibitors and were found to be in euglycemic DKA after bariatric surgery [8, 10–12]. The time interval for the development of euglycemic DKA in the patients we presented ranged from within one-week post-op to approximately one month. A case series on patients post-CABG showed that the onset of euglycemic DKA could be as rapid as within the first 24 hours [13]. The recommendations provided by the AACE state that SGLT-2 inhibitors should be discontinued for at least 24 hours after elective surgery and not resumed until the patient has resumed a normal diet [14]. This is particularly important because bariatric surgery is becoming more common and specific guidelines for this class of antihyperglycemic drug does not exist yet. The various types of bariatric surgeries show markedly improved glycemic control in patients with less medication, likely secondary to better insulin sensitivity, and even remission of diabetes entirely [15]. The recommendation on management pre- and postoperatively should be examined closely because although euglycemic DKA as a side effect is relatively rare, patients after bariatric surgery could be at a particularly increased risk.

The adjustment in antihyperglycemic medications is crucial in the postoperative period due to decreased caloric intake and increased sensitivity; however, there is a suggestion that patients on preoperative basal insulin have an increased risk of DKA if it is stopped postoperatively [15]. The insulin was stopped postoperatively in the first case we presented, and the SGLT-2 inhibitor was continued. There is some possibility that stopping the patient's insulin pushed him further into a ketotic state. The vast shift of dietary paradigm for these patients could also be a factor in increasing their likelihood of increased ketotic state. Lastly, but importantly, none of the patients we presented in this case series resumed a regular normal diet, although they were able to tolerate oral feeds.

## 4. Conclusion

The literature shows a slight risk of euglycemic diabetic ketoacidosis in people on SGLT-2 inhibitors. The SGLT-2 inhibitors are being championed for cardiovascular and renal protection in patients, and there are even suggestions that they replace metformin as a single primary oral antihyperglycemic agent in these patients. The patients we presented developed euglycemic DKA after bariatric surgery, which shows that perhaps more research should be targeted at the prolonged postoperative course of patients on SGLT-2 inhibitors and into forming specific guidelines for their use after bariatric surgery. There is overwhelming evidence in favor of their use, but it is important to make sure that we make an effort in minimizing their side effects amongst different populations taking them.

## References

[1] Qaseem A., Barry M. J., Humphrey L. L., Forciea M. A., for the Clinical Guidelines Committee of the American College of Physicians (2017). Oral pharmacologic treatment of type 2 diabetes mellitus: a clinical practice guideline update from the American College of Physicians. *Annals of Internal Medicine*.

[2] Neal B., Perkovic V., Mahaffey K. W. (2017). Canagliflozin and cardiovascular and renal events in type 2 diabetes. *New England Journal of Medicine*.

[3] Wiviott S. D., Raz I., Bonaca M. P. (2019). Dapagliflozin and cardiovascular outcomes in type 2 diabetes. *New England Journal of Medicine*.

[4] Perkovic V., Jardine M. J., Neal B. (2019). Canagliflozin and renal outcomes in type 2 diabetes and nephropathy. *New England Journal of Medicine*.

[5] Goldenberg R. M., Berard L. D., Cheng A. Y. Y. (2016). SGLT2 inhibitor - associated diabetic ketoacidosis: clinical review and recommendations for prevention and diagnosis. *Clinical Therapeutics*.

[6] Marx N., Grant P. J., Cosentino F. (2020). Compelling evidence for SGLT2 inhibitors and GLP-1 receptor agonists as first-line therapy in patients with diabetes at very high/high cardiovascular risk. *European Heart Journal*.

[7] Sajja A. P., Dey A. K., Guha A., Elnabawi Y., Joshi A. A., Kalra A. (2019). SGLT-2 inhibitors and GLP-1 agonists: first-line therapy for diabetes with established cardiovascular disease. *Journal of Cardiovascular Pharmacology and Therapeutics*.

[8] Hassan E., Ali Mekki E., Wafic W., Karim M. (2018). SGLT2 inhibition may precipitate euglycemic DKA after bariatric surgery. *Clinical Diabetes and Research*.

[9] Ogawa W., Sakaguchi K. (2016). Euglycemic diabetic ketoacidosis induced by SGLT2 inhibitors: possible mechanism and contributing factors. *Journal of Diabetes Investigation*.

[10] Lane S., Paskar D., Hamed S., Goffi A. (2018). When guidelines Fail. *A & A Practice*.

[11] Dowsett J., Humphreys R., Krones R. (2019). Normal blood glucose and high blood ketones in a critically unwell patient with T1DM post-bariatric surgery: a case of euglycemic diabetic ketoacidosis. *Obesity Surgery*.

[12] Aminian A., Kashyap S. R., Burguera B. (2016). Incidence and clinical features of diabetic ketoacidosis after bariatric and metabolic Surgery: Table 1. *Diabetes Care*.

[13] Lau A., Bruce S., Wang E., Ree R., Rondi K., Chau A. (2018). Perioperative implications of sodium-glucose cotransporter-2 inhibitors: a case series of euglycemic diabetic ketoacidosis in three patients after cardiac surgery. *Canadian Journal of Anesthesia*.

[14] Handelsman Y., Henry R. R., Bloomgarden Z. T. (2016). American Association of Clinical Endocrinologists and American College of endocrinology position statement on the association of Sglt-2 inhibitors and diabetic ketoacidosis. *Endocrine Practice*.

[15] Mulla C. M., Baloch H. M., Hafida S. (2019). Management of diabetes in patients undergoing bariatric surgery. *Current Diabetes Reports*.

